# Soil Mobility and Residual Effects of Herbicides Applied on Corn Straw

**DOI:** 10.1021/acsomega.5c06446

**Published:** 2026-03-04

**Authors:** Rita de Cássia Silva, Lucas Rêgo Mendonça Marinho, Amanda de Moraes Azevedo Pereira, Paulo Sérgio Fernandes das Chagas, Ana Beatriz Rocha de Jesus Passos, Daniel Valadão Silva, Camila Ferreira de Pinho

**Affiliations:** † 67825 Federal Rural University of Rio de Janeiro, Departament of Crop Science, BR 465—Km 7, Seropédica 23897-000, Brazil; ‡ 74384 Federal University of the Semi-Arid Region, Departament Agronomy and Forestry Science, 572 Francisco Mota Avenue, Mossoró 59625-900, Brazil

## Abstract

The efficacy of herbicides is directly related to its availability in biological targets. The presence of straw cover on the soil, coupled with dry period conditions following pre-emergence herbicide application, has raised concerns regarding pre-emergence herbicide efficacy, mainly because of the increased potential for herbicide retention and degradation. This research aimed to elucidate how different levels of corn straw and dry periods impact the mobility and residual activity of key pre-emergence herbicides used in cultivation systems with soil coverage. The results showed that diclosulam and sulfentrazone maintained their mobility and efficacy in controlling *Commelina benghalensis* L., regardless of straw cover or dry periods. However, the residual activity of diclosulam was slightly reduced by straw, while sulfentrazone remained effective 42 days after herbicide application. In contrast, the presence of straw negatively affected the mobility and efficacy of flumioxazin and S-metolachlor, reducing their effectiveness in controlling *C. benghalensis* L, regardless of dry periods. The residual activity was also negatively affected by straw on the soil, particularly in the case of flumioxazin. Herbicides with greater stability under straw and dry conditions, such as diclosulam and sulfentrazone, exhibit more consistent performance, while flumioxazin and S-metolachlor molecules require complementary management strategies to maintain efficacy. Thus, farmers should consider the amount of straw on the soil and the possibility of dry periods when selecting pre-emergence herbicides. From an environmental perspective, the distinct physicochemical properties of the evaluated herbicides determine their mobility, persistence, and potential risk of contamination through leaching and surface runoff. Understanding these interations supports the development of integrated weed management and environmental protection strategies in agricultural systems conservation.

## Graphical abstract

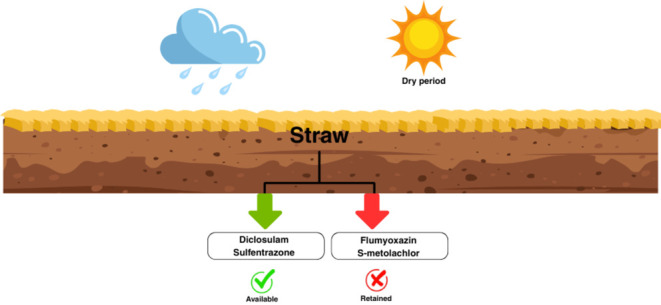

## 1 Introduction

In Brazil, the climatic conditions favor crop rotation systems, with the soybean/corn system being the most prominent. In this system, most farmers adopt no-till practices, emphasizing minimal soil disturbance and the retention of crop residues in the field. The presence of crop residues in the soil offers numerous benefits such as moisture retention and reduced erosion. However, it is noteworthy that the presence of crop residues in the soil may adversely affect the efficacy of herbicides applied in pre-emergence.^1^

The application of pre-emergence herbicides primarily aims to manage seed germination and plant emergence, thereby preventing their establishment in cultivated areas. These herbicides are applied to the soil, exhibiting residual activity that controls weeds (target species) for an extended period while maintaining selectivity toward the crops within the production system. This approach allows for alternating and combining different modes of action across seasons, reducing the selection pressure for resistance. When integrated with postemergence herbicides, it opens weed management options.^2^ The adoption of pre-emergence herbicides is increasing in Brazil and other countries due to the challenges associated with controlling and resistant weeds selected over recent decades, with glyphosate being only one of many examples of resistance cases across different weed species.^3,4^

The efficacy of pre-emergence herbicides is determined by their physicochemical properties, as well as by edaphoclimatic conditions and the crop production system adopted.^5–7^ In the no-till system, the presence of crop residue on the soil surface, resulting from crop rotation, plays a critical role in herbicide behavior. This residue acts as a physical and chemical barrier, hindering the mobility of herbicides to the soil and retaining part of the applied product through sorption processes, which can alter its availability and soil dynamics.^8^ Rainfall or irrigation following herbicide application can directly transport them from the crop residue to the soil, ensuring the efficacy of the herbicides applied in the area.^1^

However, during dry periods, intercepted herbicides become more susceptible to losses through sorption,^9^ photodegradation,^10^ volatilization,^9^ or microbial degradation,^11^ reducing the amount available for absorption by weed seeds.^12^ Herbicide retention in the soil and crop residue is strongly influenced by the solubility and polarity of the compounds, as well as by the amount of residue present on the soil surface and the interval between herbicide application and rainfall events.^13,14^

Diclosulam, flumioxazin, sulfentrazone, and S-metolachlor have been the primary options for pre-emergence application in soybean and corn crops in Brazil, as well as for weed management during the offseason to reduce the soil seed bank of challenging species. However, Brazilian farmers have reported variations in the efficacy of certain pre-emergence herbicides. Our hypothesis is that the presence and quantity of crop residue, coupled with increasing dry periods, may alter the mobility, persistence, and bioavailability of pre-emergence herbicides, compromising their efficacy due to increased retention. Therefore, the objectives of this study were (1) to assess the influence of corn straw and dry periods on the mobility of diclosulam, flumioxazin, S-metolachlor, and sulfentrazone, and (2) to determine the residual effect.

## 2 Materials and Methods

### 2.1 Experimental Conditions

The soil used in the research was collected from an area with no history of herbicide use located at Federal Rural University of Rio de Janeiro (22°45′44.5″S 43°41′57.0″W), and samples were taken from the surface layer of the soil (0–20 cm), air-dried, and sieved through a 2 mm mesh. The physical and chemical attributes of the soil were analyzed at the Soil-Plant Interactions Study Laboratory of the Federal Rural University of Rio de Janeiro (Table 1).

**Table 1:** Physicochemical Analysis of Soil in an Area with No History of Herbicide Use

**granulometry**(g kg^**–1**^ **ADSS** ^a^ **)**		
**sand**	**silt**	**clay**
839	92	69

	**exchangeable cations**(Cmol_**c**_ **/dm** ^**3**^ **)**								**Corg** ^d^
**pH**(H_**2**_ **O)**	**Ca**	**Mg**	**K**	**Na**	**S** ^b^	**Al**	**H+Al**	**CEC** ^c^	**%**
5.19	1.2	1	0.01	0	2.7	0.05	0.6	3	1.85

a ADSS: air-dried soil samples.

b S: exchangeable bases summary (Ca + Mg + Na + K).

c CEC: (S + H + Al).

d Corg: organic carbon.

The experimental units consisted of polyethylene pots (22 cm in diameter) with a capacity of 5 L filled with previously characterized soil. The plant residues (corn straw) used in the experiments were collected after the natural drying of plants grown in the Major Crops Sector of the Federal Rural University of Rio de Janeiro. The residues were then transported to the laboratory, and the material was manually cut into fragments approximately 2 × 5 cm in size. These residues were then transported to the laboratory for subsequent homogenization and weighing.

### 2.2 Experimental Design and Analyzed Variables

#### 2.2.1 Assessment of Herbicide Mobility and Residual Activity

Four experiments were conducted by using a randomized complete block design with four replications. One experiment was conducted for each herbicide: diclosulam (Spider 840, 35 g ai ha^–1^, WG, Dow Chemical Company), flumioxazin (Flumyzim 500, 60 g ai ha^–1^, WP, Sumitomo Chemical CO), S-metolachlor (Dual Gold, 1800 g ai ha^–1^, EC, Syngenta), and sulfentrazone (Boral 500, 600 g ai ha^–1^, SC, FMC Corporation), and their physicochemical characteristics are presented in Table 2. These experiments were repeated to determine the amount of herbicide retained in the straw and soil under the same experimental conditions except for the indicator plant cultivation.

**Table 2:** Physicochemical Characteristics of the Analyzed Herbicides Regarding Their Acid/Base Ionization Constant (p*K*_a_), Solubility, and Lipophilicity (K_ow_)

**herbicides**	**p** *K*_**a**_	**solubility**(mg L^**–1**^ **)**	**K_ow_**
diclosulam	4.09	117 to 124	1.42
flumioxazin	nonionic	1.79	2.55
S-metolachlor	nonionic	480	3.05
sulfentrazone	6.56	780	0.99

The treatments of each experiment were arranged in a 3 × 3 + 2 factorial design with factor A corresponding to levels of corn straw added to the soil surface (0, 50, and 100%—equivalent to 3 t ha^–1^), and factor B consisting of precipitation periods (1, 10, and 20 days after herbicide application—DAHA), in addition to control groups without herbicide application, both in the presence and absence of straw.

Precipitation of 20 mm was simulated in each experimental unit by using an irrigation system with a flow rate of 1.9 L min^–1^. This simulation was conducted at 1, 10, and 20 days after herbicide application (DAHA) (depending on the treatment). Following this procedure, the pots were subjected to daily irrigation of 4 mm solely for the maintenance of the bioassay, ensuring that soil moisture remained close to field capacity, with average temperatures during the experimental period ranging from approximately 20 to 30 ± 5 °C.

Herbicide mobility was assessed by using the bioassay method. *Commelina benghalensis* L. (Benghal dayflower) was employed as an indicator plant for the studied herbicides. In each pot, 50 *C. benghalensis* seeds were sown in dry soil and covered with the amount of straw corresponding to each treatment. Subsequently, herbicide application was conducted. Seeds were planted to a depth of approximately 2 cm.

The herbicide application was conducted using a pressurized CO_2_ backpack sprayer operating at a pressure of 2.8 Kpa, equipped with a spray boom featuring two TT11002 nozzles spaced at 50 cm intervals, and applying a spray volume of 150 L ha^–1^. Meteorological data at the time of application were recorded as 20.2 °C temperature, 88% humidity, and wind speed of 1.9 m s^–1^, according to the National Institute of Meteorology database (INMET, 2021).

At 21 days after each simulated precipitation event, visual control ratings were assigned to *C. benghalensis*, with 100% indicating complete control and 0% indicating the absence of symptoms. At 42 days after herbicide application, plant harvesting was conducted, and the aboveground dry mass (ADM) of *C. benghalensis* was weighted. The plants were cut at ground level, placed in paper bags, and subjected to a drying process in a circulating air oven for 72 h at 60 °C and weighted in analytical scale.

Furthermore, immediately following harvest, 50 *C. benghalensis* seeds were sown to assess the residual activity of herbicides on the weed. At 21 and 35 days after sowing for residual (DASR) control of *C. benghalensis*, the visual control percentage was assessed using the same procedure as previously described.

#### 2.2.2 Extration and Quantification of Herbicides in Soil and Corn Straw

Soil and straw collection for herbicide residual quantification was conducted 42 days after application. Cylinders with a 2.5 cm diameter and 10 cm length and a 4 cm diameter and 3 cm length were used for soil and straw collections, respectively. Two small samples were collected from different locations in the pots and homogenized throughout the collections. The samples were transferred to hermetic plastic bags and stored in a freezer (−20 °C) for subsequent analysis. The extraction of herbicides present in the soil and straw samples was performed using the QuEChERS method,^15^ with some modifications. Initially, 5 g of air-dried soil or straw were weighed into 50 mL Falcon tubes. Then, 10 mL of acetonitrile, 100 μL of acetic acid, and 2 mL of distilled water were added. The tubes were shaken vertically for 15 min and subsequently subjected to an ultrasonic bath for 20 min. After this step, 1 g of NaCl and 2 g of MgSO_4_ were added to each tube, followed by vortexing for 1 min and centrifugation at 3600 rpm for 5 min. An aliquot of the supernatant was then collected, filtered through a 0.22 μm nylon membrane, and stored in vials for analysis by ultrahigh-performance liquid chromatography coupled with tandem mass spectrometry (UHPLC-MS/MS).

### 2.5 Chromatographic and Mass Spectrometry Conditions

The Ultra-High-Performance Liquid Chromatography (UHPLC) equipment Nexera X2 (Shimadzu, Tokyo, Japan) was equipped with two LC-30AD pumps, a DGU-20A_5R_ degasser, a Sil-30AC autosampler, a CTO-30AC column oven, and a CBM-20A controller. Separation occurred on a Restek column (Pinnacle DB AQ C18, 50 × 2.1 mm, 1.9 μm particles). Chromatographic operation was carried out with a flow rate of 0.20 mL min^–1^, an injection volume of 5 μL, and sampler and column oven temperatures set at 15 and 40 °C, respectively. Mobile phase A consisted of HPLC-grade water with 0.1% formic acid, and mobile phase B was HPLC-grade acetonitrile. The elution was isocratic for sulfentrazone, diclosulam, and S-metolachlor, and the flow contained 70% of mobile phase B, while for flumioxazin, the flow was composed of 85% of mobile phase B. The triple quadrupole mass spectrometer LCMS-8040 series (Shimadzu, Tokyo, Japan) with electrospray ionization (ESI) source was operated in both positive and negative ionization modes.

Regarding the method performance parameter, the recovery percentages obtained in soil and crop residue at the analyzed concentrations for sulfentrazone, diclosulam, flumioxazin, and S-metolachlor ranged between 70 and 120%, with an RSD equal to or less than 20%. The presented results (Table 3) confirm the effectiveness of the method in extracting herbicide residues from the matrices.^16^

**Table 3:** Percentual of Herbicide Recovery

		concentration					
		10 μg/kg		100 μg/kg		500 μg/kg	
substance	matrix	recovery (%)	RSD^a^ (%)	recovery (%)	RSD (%)	recovery (%)	RSD (%)
sulfentrazone	soil	91.21	3.35	105.62	3.98	91.82	1.81
	straw	108.43	7.06	113.87	4.97	108.44	4.26
diclosulam	soil	94.70	1.20	91.11	1.16	95.57	1.95
	straw	110.89	3.91	113.25	9.13	103.45	4.85
flumioxazin	soil	93.71	7.55	97.08	2.40	91.40	6.36
	straw	112.16	3.17	104.22	2.85	107.68	2.77
S-metolachlor	soil	86.09	6.94	92.69	2.99	94.05	0.43
	straw	106.42	8.65	111.24	5.74	107.15	2.20

a RSD: relative standard deviation

### 2.6 Data Analysis

The data were subjected to analysis of variance (ANOVA) using R Studio (2023.03.0 Build 386), and means were separated using Tukey HSD (Honestly Significant Difference) test at α = 0.05. The figures were created by using the SigmaPlot program (version 12.5 for Windows). The aboveground dry mass data were transformed into a percentage of dry mass reduction (DMR) compared to the control based on eq 1.^17^ The amount of residual herbicide (QHR) present in the soil and straw was determined according to eq 2.

1 $$DMR=100-\frac{\left(treated\;plant\;dry\;mass\times 100\right)}{untreated\;plant\;dry\;mass}$$

2 QHR=CXM

where QHR (μg) represents the amount of residual herbicide, *C* (μg kg^–1^) is the herbicide concentration in the soil or straw, and *M* (kg) is the mass of soil or straw used per experimental unit.

## 3 Results

### 3.1 Mobility of Pre-emergence Herbicides

Interaction occurred between the precipitation periods and levels of straw for all herbicides in all variables analyzed in the study.

Diclosulam resulted in up to 90% of the control *C. benghalensis* in the absence of straw in all precipitation periods (Figure 1A). In the presence of straw at 1.5 and 3 t ha^–1^, the control was 80 and 95%, respectively, regardless of the dry period. The reduction in the *C. benghalensis* dry mass exceeded 83% in all evaluated treatments (Figure 2A).

**Figure 1:**
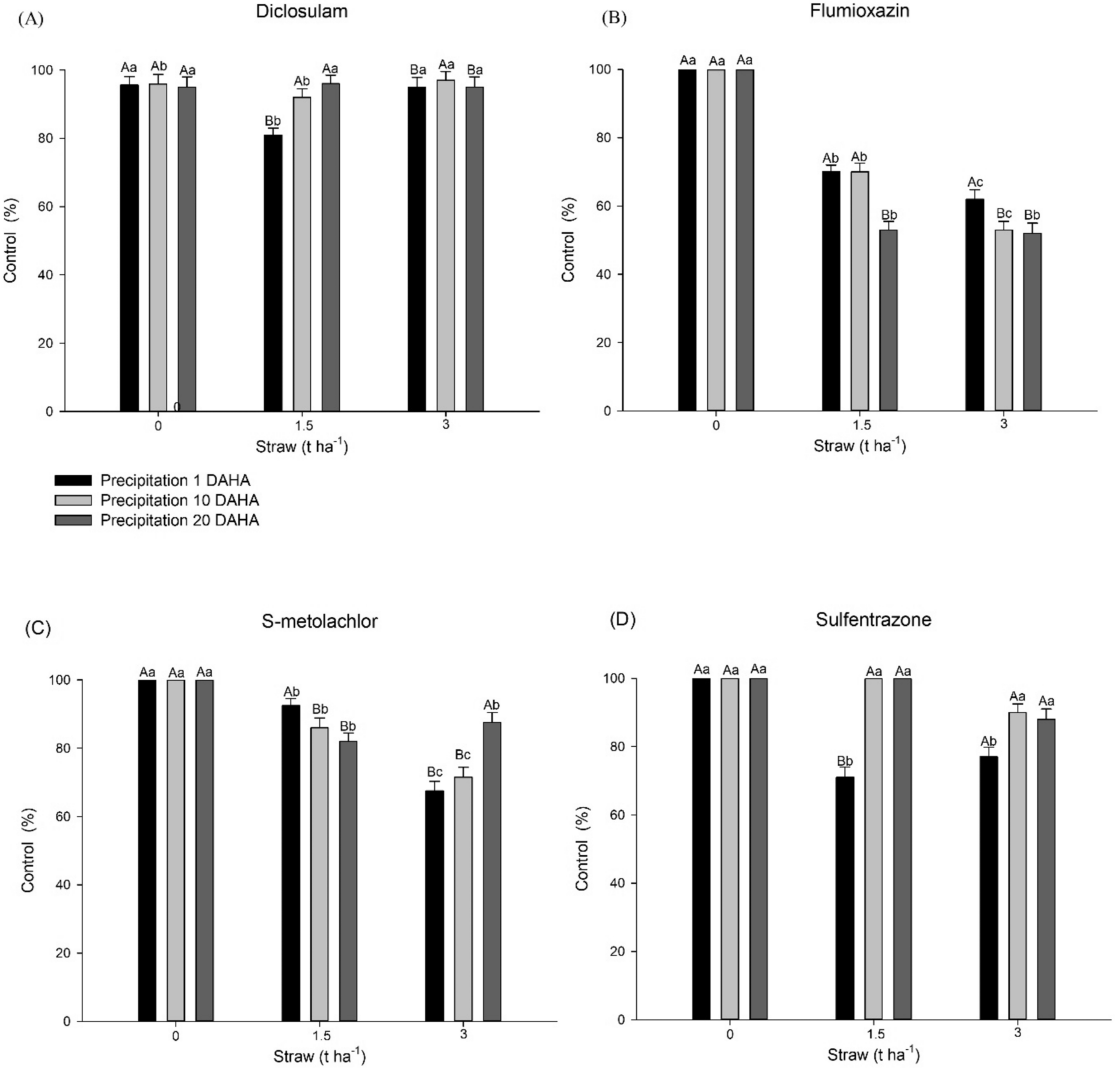
*C. benghalensis* L. control (%) at 21 days after each simulated precipitation event following diclosulam (35 g ai ha^–1^) (A), flumioxazin (60 g ai ha^–1^) (B), S-metolachlor (1800 g ai ha^–1^) (C), and sulfentrazone (600 g ai ha^–1^) (D) application influenced by precipitation periods and different amounts of corn straw. Means followed by the same lowercase letters between precipitation periods and uppercase letters between different straw amounts do not differ significantly from each other using the Tukey HSD test (*P* ≤ 0.05).

**Figure 2:**
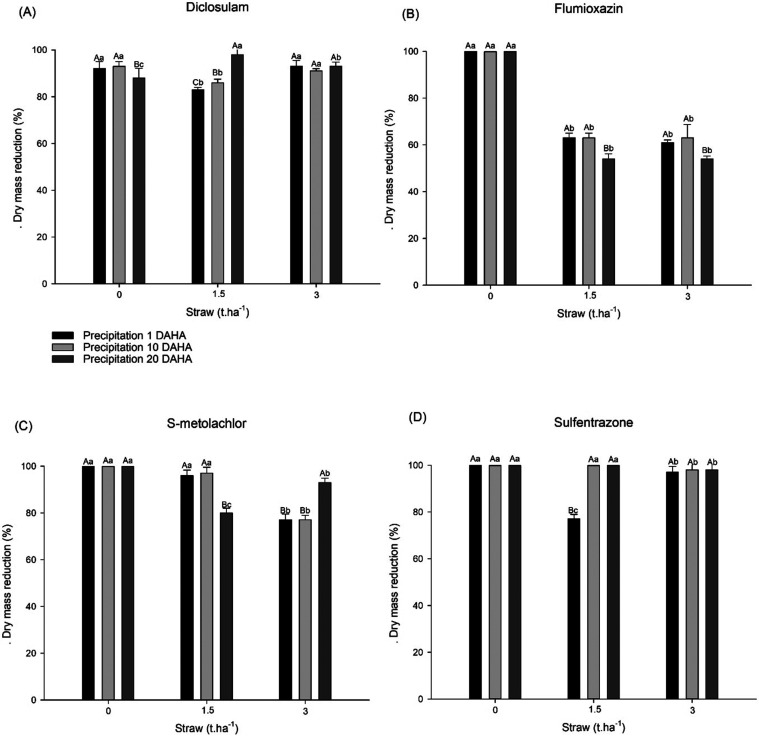
Dry mass reduction (%) of *C. benghalensis* L. caused by diclosulam (35 g ai ha^–1^) (A), flumioxazin (60 g ai ha^–1^) (B), S-metolachlor (1800 g ai ha^–1^) (C), and sulfentrazone (600 g ai ha^–1^) (D) at 21 days after herbicide application compared to untreated plants and influenced by precipitation periods and different amounts of corn straw. Means followed by the same uppercase letters in columns and lowercase letters in lines do not differ significantly from each other using the Tukey HSD test (*P* ≤ 0.05).

In the absence of straw, flumioxazin exhibited 100% control of *C. benghalensis* in all precipitation periods (Figure 1B). Nevertheless, the control was reduced to approximately 75% in the presence of straw at 1.5 t ha^–1^ for precipitation periods at 1 and 10 DAHA, and to 55% at 20 DAHA. A decrease in the control of approximately 60% was observed in the highest straw level (3 t ha^–1^). In treatments without straw, a 100% reduction in dry mass was observed, regardless of precipitation. In the presence of crop residue, regardless of the quantity, rainfall simulated at 20 DAHA resulted in the lower reduction in dry mass, reaching 54%, and did not exceed 63% in the other evaluated precipitation periods (Figure 2B).

In the absence of straw, S-metolachlor exhibited 100% control regardless of rainfall (Figure 1C). For treatments with 1.5 t ha^–1^ of straw, the control ranged between 80 and 90%, with the highest efficacy observed for rain occurring 1 day after application, gradually decreasing in subsequent precipitation events. In the presence of 3 t ha^–1^ of straw, the control did not exceed 72% in the 1 and 10-day periods and reached 87% in the 20-day period without rainfall. A decrease in dry mass was observed in treatments with 3 t ha^–1^ of straw, ranging from 77 to 93%, and with 1.5 t ha^–1^ of straw, ranging from 80 to 100%. In the absence of straw, the greatest reduction was achieved, reaching 100% (Figure 2C).

In the absence of straw, complete control of *C. benghalensis* was achieved through the application of sulfentrazone in all precipitation periods (Figure 1D). Conversely, in the presence of crop residue, regardless of intensity, satisfactory control (above 80%) was observed only when precipitation occurred 10 and 20 days after application. Additionally, for 1.5 t ha^–1^ of crop residue, the control was higher in the periods of 10 and 20 days of drought after application, highlighting the impact of residue intensity on herbicide efficacy (Figures 1D). The aerial dry mass reduction data align with the control results observed at 21 days after each simulated precipitation. A decrease in the mass of 77% was observed in treatments containing straw that received rain 1 day after herbicide application; such a decrease was less pronounced in the treatment containing 1.5 t ha^–1^ of straw (Figure 2D).

### 3.2 Residual Activity of Pre-emergence Herbicides

In the absence of straw and with 20 days without rainfall, 88% control of *C. benghalensis* under the influence of diclosulam was achieved. In contrast, during the 1- and 10-day periods, the control range for *C. benghalensis* was approximately 73 and 82%, respectively (Figure 3A). In the presence of 1.5 t ha^–1^ of straw, it was observed that over the extended period of 20 days without rainfall, the control was 80%, while in the other periods of 1 and 10 days, the control did not exceed 75%. With 3 t ha^–1^ of straw on the soil, the control was 89% during the dry periods of 10 and 20 days, and in the 1-day period, the control was 71%.

**Figure 3:**
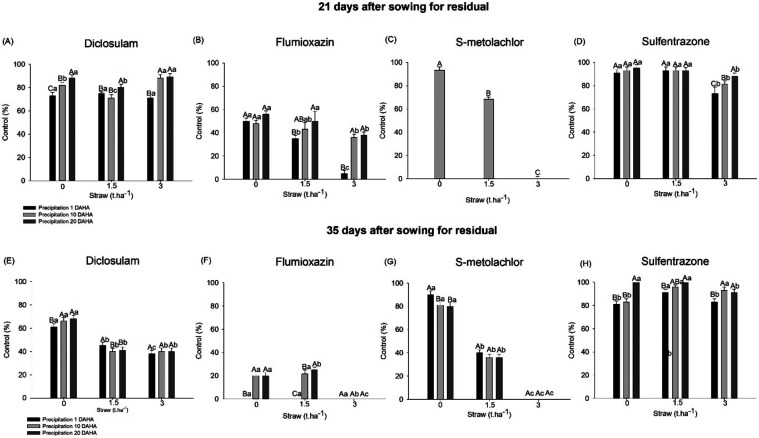
*C. benghalensis* L. control at 21 and 35 days after sowing for residual activity for diclosulam (35 g ai ha^–1^) (A and E), flumioxazin (60 g ai ha^–1^) (B and F), S-metolachlor (1800 g ai ha^–1^) (C and G), and sulfentrazone (600 g ai ha^–1^) (D and H) influenced by precipitation periods and different amounts of corn straw. Means followed by the same uppercase letters between precipitation periods and lowercase letters between straw amount do not differ significantly from each other using the Tukey HSD test (*P* ≤ 0.05).

At 35 days after sowing for residual (77 days after herbicide application), there was a reduction in the control in all treatments (Figure 3E). In the absence of straw, the control efficacy of diclosulam did not exceed 68%, regardless of the precipitation period. In treatments with straw on the soil, the control did not surpass 45% in all of the evaluated precipitation periods (Figure 3E).

For the residual effect of flumioxazin herbicide in the absence of straw, at 21 DASR, the obtained control (50%) was similar for all precipitation periods (Figure 3B). In the presence of straw, superior control was obtained when rain occurred 20 days after herbicide application. Furthermore, with 3 t ha^–1^ of straw on the soil, the control was significantly lower than that observed with 1.5 t ha^–1^ of straw, being 5 and 38%, respectively, for rainfall 1 and 20 days after herbicide application (Figures 3B). At 35 DASR, diclosulam exhibited unsatisfactory control (below 20%) among all treatments, as evidenced by the normal emergence and development of all *C. benghalensis* plants (Figure 3F).

At 21 DASR, there was no interaction between the factors for the herbicide S-metolachlor, but there was a significant difference due to the presence of straw (Figure 3C). The obtained controls were 92, 65, and 0%, respectively, in the levels of straw of 0, 1.5, and 3 t ha^–1^. At 35 DASR, there was an interaction between the precipitation periods and straw levels (Figure 3G). In the absence of straw, there was a difference between precipitation periods, with a control of 90% for the 1-day period without rain and 80% at 20 days without rain.

The control obtained for all treatments with sulfentrazone was above 80%, where the highest control was achieved for longer rain-free periods (Figure 3D). At 35 DASR, in the absence of straw, the control was 81 and 83% for the 1 and 10 days without rain periods, respectively, and 100% for the 20 days without rain (Figure 3H).

### 3.3 Quantification of Herbicides in a Straw and Soil

The ratio between the herbicide retained in the straw and the herbicide present in the soil was not calculated since the amount retained in the straw was low. Regardless of the amount of straw, less than 1% of the applied herbicide remained in the straw after rainfall simulation.

The highest straw level (3 t ha^–1^) contributed to the increased retention of diclosulam compared to 1.5 t ha^–1^ of straw (Table 4). In the period of 1 day without rainfall for 1.5 t ha^–1^ of straw, the herbicide transposition was lower, equivalent to 0.0760 μg, compared with the periods of 10 and 20 days without rain. For the straw level of 3 t ha^–1^, the lower transposition was observed in the periods of 1 and 10 days without rain compared to the period of 20 days without rain. In the analyses of total herbicide reaching the soil, regardless of straw levels on the soil, a higher herbicide amount was observed in treatments that received rain 1 day after application.

**Table 4:** Diclosulam Quantification in Corn Straw and Soil after Rainfall Simulation at 42 Days after Herbicide Application^a^

	straw (μg)		soil (μg)		
period (days)	1.5	3	0	1.5	3
1	0.0760^Ab^	0.1601^Aa^	151.70^Aa^	69.70^Ab^	53.83^Ac^
10	0.0446^Bb^	0.1591^Aa^	132.11^Ba^	52.21^Bb^	29.66^Bc^
20	0.0396^Bb^	0.1199^Ba^	100.53^Ca^	34.97^Cb^	22.50^Bc^
*F*_period × soil_ = 17.23			*F*_period × straw_ = 4.97		
CV (%) = 9.09			CV (%) = 7.3		

a The herbicide amount of 159.60 μg was calculated for each pot. Means followed by the same uppercase letters in the columns and lowercase letters in the rows do not differ significantly by the Tukey HSD test (*p* ≤ 0.05).

Conversely, in the presence of straw, the amount of herbicide found in the soil was less than 50% of that obtained in treatments without straw (Table 4). Still, the reduced amount of herbicide that reached the soil ensured control of *C. benghalensis* like treatments without straw, as evident in Figure 1A.

The lowest concentration of flumioxazin was obtained when precipitation occurred 21 days after herbicide application regardless of the level of straw (Table 5). Additionally, in the straw level of 3 t of ha^–1^, the concentration was approximately 2.5 times higher than that found in the 1.5 t of ha^–1^ level of straw. Furthermore, in soil treatments covered with straw, a reduced herbicide concentration in the soil was observed since the shortest rain time.

**Table 5:** Flumioxazin Quantification in Corn Straw and Soil after Rainfall Simulation at 42 Days after Herbicide Application^a^

	straw (μg)		soil (μg)		
period (days)	1.5	3	0	1.5	3
1	0.1766^Ab^	0.4400^Aa^	433.54^Aa^	278.99^Ab^	232.93^Ac^
10	0.1466^Ab^	0.4133^Aa^	284.75^Ba^	191.24^Bb^	172.60^Bb^
20	0.0966^Bb^	0.2433^Ba^	194.90^Ca^	150.90^Cb^	135.19^Cb^
*F*_period × soil_= 16.95			*F*_period × straw_ = 14.04		
CV (%) = 13.55			CV (%) = 7.41		

a The herbicide amount of 455.40 μg was calculated for each pot. Means followed by the same uppercase letters in the columns and lowercase letters in the rows do not differ significantly by the Tukey HSD test (*p* ≤ 0.05).

In the treatment containing 1.5 t ha^–1^ of straw, a higher translocation of S-metolachlor was observed when precipitation occurred at 1 day after application, while for the periods of 10 and 20 days without rain, a higher herbicide concentration was found in the straw (Table 6). The same trend was observed in the treatment containing 3 t ha^–1^ of straw. In the soil, the amount of S-metolachlor applied corresponded to 7294.68 μg, where in the absence of straw a total of 6071.37 μg was recovered in the period of 1 day without rain. This recovery decreased with the dry period, reaching 4183.70 μg when rain occurred 20 days after application. In the presence of straw, the response to precipitation was similar; however, the values were lower than those found in the absence of straw for all treatments. In levels of straw of 1.5 and 3 t ha^–1^, respectively, 5607.95 and 3039.49 μg of the herbicide were recovered in the period of 1 day without rain (Table 6).

**Table 6:** S-metolachlor Quantification in Corn Straw and Soil after Rainfall Simulation 42 Days after Herbicide Application^a^

	straw (μg)		soil (μg)		
period (days)	1.5	3	0	1.5	3
1	0.0625^Bb^	1.5452^Ba^	6071.37^Aa^	5607.95^Ab^	3039.49^Ac^
10	0.3805^Ab^	1.8674^Aa^	5463.30^Ba^	3456.04^Bb^	2158.09^Bc^
20	0.3748^Ab^	1.0631^Ca^	4183.70^Ca^	2585.42^Cb^	1709.03^Cc^
*F*_period × soil_= 28.29			*F*_period × straw_ = 431.90		
CV (%) = 14.45			CV (%) = 1.13		

a The herbicide amount of 7294.68 μg was calculated for each pot. Means followed by the same uppercase letters in the columns and lowercase letters in the rows do not differ significantly by the Tukey HSD test (*p* ≤ 0.05).

The data for quantification of the herbicide sulfentrazone are presented in Table 7. For both straw levels evaluated (1.5 and 3 t ha^–1^), there was greater transposition of the herbicide (lower retention in the straw) in the period of 20 days without rain, and greater retention in the period of 1 day without rain, supporting the observed control data (Figure 1D). In the soil, when 1138.5 μg of sulfentrazone was applied without straw, more herbicide (981.64 μg) was recovered on the day 1 without rain treatment, while a lower amount (798.65 μg) was recovered during the extended dry period of 20 days. The presence of straw led to a decrease in the detected herbicide values in the soil, particularly in straw with higher intensity (Table 7).

**Table 7:** Sulfentrazone Quantification in Corn Straw and Soil after Rainfall Simulation at 42 Days after Herbicide Application^a^

	straw (μg)		soil (μg)		
period (days)	1.5	3	0	1.5	3
1	0.0625^Bb^	1.5452^Ba^	6071.37^Aa^	5607.95^Ab^	3039.49^Ac^
10	0.3805^Ab^	1.8674^Aa^	5463.30^Ba^	3456.04^Bb^	2158.09^Bc^
20	0.3748^Ab^	1.0631^Ca^	4183.70^Ca^	2585.42^Cb^	1709.03^Cc^
*F*_period × soil_ = 28.29			*F*_period × straw_ = 431.90		
CV (%) = 14.45			CV (%) = 1.13		

a The herbicide amount of 1138.5 μg was calculated for each pot. Means followed by the same uppercase letters in the columns and lowercase letters in the rows do not differ significantly by the Tukey HSD test (*p* ≤ 0.05).

## 4 Discussion

Generally, ALS-inhibiting herbicides, such as diclosulam, exhibit important characteristics related to their mobility and persistence in the soil, with their behavior being strongly influenced by soil organic matter content and moisture levels. Microbial degradation is the primary pathway for the breakdown of these herbicides, with degradation by light being negligible.^18,19^

The physicochemical properties of herbicides, such as octanol–water partition coefficient (K_ow_), acid dissociation constant (p*K*_a_), solubility, and soil organic carbon partition coefficient (K_oc_) are key determinants of mobility, persistence, and bioavailability in soil and crop residues. These parameters, combined with soil pH, texture, and moisture, define the extent of herbicide movement and degradation in conservation systems.^20–22^

Diclosulam presents a low octanol–water partition coefficient (K_ow_ = 1.42) (Table 2), indicating limited lipophilicity, and a low organic carbon partition coefficient (K_oc_), suggesting weak sorption to soil organic matter. Combined with its moderate solubility, these characteristics benefit herbicide’s mobility through crop residues following precipitation.^23^ Diclosulam at a rate of 25.2 g ai ha^–1^ in sorghum residue, followed by a 30 mm rainfall, proved to be effective in leaching into the soil up to 35 days after application, ensuring efficiency in the controlling *Ipomea grandifolia* and *Sida rhombifolia*.^23^ In addition, its low distribution coefficient (*K*_d_ = 1.1 L kg^–1^) indicates a limited tendency to bind to soil particles, enhancing availability in the soil solution.^24^ In this experiment, the soil had a pH of 5.19, higher than the herbicide’s p*K*_a_ of 4.09, indicating predominance in the anionic form, ensuring lower sorption and persistence and greater availability for movement in the soil profile.

The results indicate that diclosulam’s performance was not affected by the presence of straw, likely due to its ability to move through crop residues. Although dry periods slightly reduced the herbicide concentration in the soil, enough remained available to ensure effective control of *C. benghalensis* regardless of the straw level. The absence of photodegradation may also contributes to this process. Even during rainless periods, where the product remains exposed to sunlight after application, it is not degraded, remaining available to be transported to the soil after rainfall.^25^

In the residual activity experiment, under a soil texture of 83.9% sand, 6.9% clay, and a pH of 5.19, leaching may occur easily. Lixiviation losses are favored in sandy soils or those with lower organic matter content,^21^ this may lead to groundwater contamination, a critical environmental concern.^26^ The soil pH may have contributed to diclosulam degradation since available molecules are in an anionic form, becoming more polar and soluble, and thus more susceptible to microbial activity.^22^ Conditions favoring microbial activity include high temperatures, moisture, and aeration.^27^ The lower control values may be associated with the reduced availability of diclosulam in the soil solution due to its degradation (Figure 2E).

Flumioxazin, characterized by low solubility (1.79 mg L^–1^) and high K_ow_ (2.55), (Table 2), indicates low mobility in crop residues. Additionally, flumioxazin is susceptible to photodegradation,^28^ which may explain the slightly lower control in precipitations 20 days after application, as the product was exposed to sunlight for a longer period.

Applying flumioxazin on corn and oat straw for controlling *Brachiaria decumbens*, *Bidens pilosa*, *Sida rhombifolia*, *Ipomoea nil*, *Ipomoea grandifolia*, and *Digitaria* spp. after 1, 15, 30, and 60 rainless days resulted in reduced effectiveness when the time gap between application and rainfall exceeded 30 days, aligning with findings from this study.^28^ The decrease in *C. benghalensis* control between straw amounts of 1.5 and 3 t ha^–1^ in soil may be attributed to herbicide degradation induced by light until the rainfall simulation. The time during which the herbicide persists in the straw without rainfall might contribute to heightened degradation of the molecule through photolysis, given the low microbial activity in dry straw study.^28–30^

These results indicate that for the herbicide flumioxazin, 77 days after application, the straw retained a significant portion of the herbicide, preventing it from reaching enough to exert a prolonged residual activity in the soil.

The adsorption and desorption characteristics of flumioxazin were investigated in seven soils in the southern United States. The study found that soils with very low clay and organic matter content exhibited the least adsorption of flumioxazin, with a sorption capacity (*K*_f_) of 0.4.^31^ The soil used in our study consists of 83.9% sand, 6.9% clay, and 1.85% *C*_org_, potentially influencing the herbicide’s low adsorption in the soil and resulting in a diminished residual effect. Furthermore, the herbicide’s low *K*_d_ and *K*_f_ values suggest limited mobility within the soil.^31,32^ The sandy soil used in this study may have contributed to the low adsorption and reduced residual activity.

S-metolachlor, with moderate solubility (480 mg L^–1^) and high K_ow_ (3.05), demonstrated a reduction in control levels proportional to the increase in straw content. When S-metolachlor was applied to sugarcane straw under different precipitation intervals for *Panicum maximum* control, a higher herbicide efficacy was observed in the absence of straw.^33^ The sorption of S-metolachlor in the soil is positively correlated with the organic matter and clay content, with a variation in organic matter from 0.9 to 5.7% resulting in an approximately 6-fold increase in the sorption coefficient (*K*_d_) of S-metolachlor.^34^ In this study, for soils containing 1.85% *C*_org_, 6.9% clay, and 83.9% sand, the sorption of the herbicide S-metolachlor in the soil was not favored. This indicates that the higher interval between herbicide application and rainfall resulted in lower S-metolachlor concentrations in the soil (Table 6).

The residual activity of S-metolachlor was significantly influenced by the increase in the straw content on the soil, evidenced by the severe decrease in *C. benghalensis* control with the higher amount of straw (Figure 3G) added to a high K_ow_ (794 at 25 °C) and high *K*_d_ (1869 mLg^–1^), which gives it a greater capacity for retaining organic carbon and plant residues. Studies evaluating the residual activity of S-metolachlor in sandy and clayey soils stated that in sandy soil samples, S-metolachlor controlled the indicator plants by 80% up to 52 days after the application, while in clayey soil, it exhibited control up 96% up to 80 days after application.^35^

On the other hand, herbicides with high retention in straw, such as flumioxazin and S-metolachlor, are more susceptible to photodegradation and volatilization before reaching the soil, which reduces their residual activity but also their leaching potential.^30,32^ However, surface runoff losses may occur in the event of heavy rainfall shortly after application, as the straw containing herbicide residues can be washed away, contributing to the contamination of surface water bodies.^36^

Sulfentrazone, in turn, show high pH-dependent solubility (780 mg L^–1^) and low K_ow_ (0.99), favoring mobility through straw and persistence in the soil.^37^ Sulfentrazone’s release from crop residues to the soil seems to be correlated with the increasing amount of rainfall after herbicide application. In a study where sulfentrazone was applied to 5 and 20 t ha^–1^ of sugarcane straw, a transposition of 77 and 64% was observed, respectively, after a 20 mm rainfall.^38^ Conversely, a 10 mm rainfall was insufficient to transpose the herbicide in the same amounts of the same residue.^39^

Simultaneously, it is important to highlight the correlation between soil pH (5.19) and the p*K*_a_ of sulfentrazone (6.56) (Table 2), where the herbicide predominantly exists in its anionic form, indicating a higher proportion of the herbicide in its nondissociated form and less availability in the soil solution. In soils with high organic matter and clay, sulfentrazone tends to be strongly adsorbed,^40,41^ justified by the higher specific surface area and available adsorption sites. The soil composition, with 83.9% sand, 6.9% clay, and 1.85% *C*_org_, creates favorable conditions for herbicide availability and activity due to low sorption in soil components and the limited electrostatic exchange surface of the sand.^29^

The high weed control percentages achieved with sulfentrazone, regardless of the amount of straw in the soil, demonstrated the straw’s lack of influence on sulfentrazone’s residual activity in the soil. Sulfentrazone has moderate mobility, poor susceptibility to photodegradation, and elevated persistence in the soil,^42,43^ which may have contributed to the elevated residual activity in *C. benghalensis* control, even when straw limited the herbicide amounts reaching the soil.

The degradation of sulfentrazone in soils used for sugarcane cultivation in Mato Grosso do Sul, considering various moisture levels, temperatures, and depths, resulted in a half-life ranging from 34 to 116 days.^44^ When examining the persistence and dissipation of sulfentrazone in dry soil, it was noted that its phytotoxic effects endured up to 182 days postapplication, with an average dissipation rate of 2.5 g ha^–1^ and a half-life exceeding 182 day.^45^ Herbicides that inhibit PROTOX (Protoporphyrinogen IX oxidase) are known for their effectiveness at low doses,^25^ which might explain why the reduction in herbicide concentration due to straw did not impede control or residual activity. Furthermore, the persistence of sulfentrazone, even at low concentrations, may raise concerns regarding its potential for bioaccumulation and its impact on soil microbiota.^46,47^

The results revealed differences in the behavior of the evaluated herbicides, depending on the presence of crop straw and the duration of dry periods. Herbicides such as diclosulam and sulfentrazone showed consistency even under adverse conditions, which reinforces their potential to compose more stable management programs in conservation systems. The greater sensitivity of flumioxazin and S-metolachlor to straw and water deficit indicates the need for complementary practices, such as adjustments in the timing of application, integration with herbicides or other mechanisms of action, or the use of cultural practices that minimize the risk of failure. This information provides important support for Brazilian farmers and those in other countries who adopt the no-until system, indicating which molecules offer greater safety of use and which require complementary management strategies to avoid control failures while also integrating this knowledge to minimize environmental risks, considering the specific physicochemical properties of each molecule and local edaphoclimatic conditions.

It is important to highlight, however, that this study was conducted under controlled conditions, which may not fully reflect the complexity and variability found in commercial agricultural areas. Edaphoclimatic variations, diversity of infesting species, and differences in management practices can alter the dynamics of the herbicides observed in the laboratory. The results presented here should be considered as an initial orientation base, which needs validation in different productive scenarios to consolidate broader technical recommendations. Future field-scale research and modeling studies may broaden the understanding of the mobility and persistence of these molecules, allowing for more precise and adapted recommendations to the realities of Brazilian agriculture.

## 5 Conclusions

The corn straw and dry periods influence the mobility and availability of flumioxazin, S-metolachlor, diclosulam, and sulfentrazone in the soil. Each molecule was affected differently based on its physicochemical characteristics.

The presence of corn straw and dry periods does not impact the arrival of diclosulam and sulfentrazone in the soil, ensuring a sufficient amount for effective *C. benghalensis* control. Diclosulam’s residual effect is mildly affected by straw, while sulfentrazone maintains ample residual efficacy for up to 77 days postapplication.

Flumioxazin and S-metolachlor exhibit similar responses under the evaluated conditions. The presence of corn straw influences the mobility of these herbicides to the soil, diminishing their efficacy regardless of the assessed precipitation periods. Moreover, the residual effect of these herbicides is nearly negligible in the presence of straw on the soil with a more pronounced impact on flumioxazin.
